# Full Circle: How ideas unfold

**DOI:** 10.1128/msphere.00485-23

**Published:** 2023-10-18

**Authors:** Aaron P. Mitchell, Michael J. Imperiale

**Affiliations:** 1 Department of Microbiology, University of Georgia, Athens, Georgia, USA; 2 Department of Microbiology and Immunology, University of Michigan, Ann Arbor, Michigan, USA

## EDITORIAL

Today we introduce a new series of minireview articles under the heading “Full Circle.” These articles are distinctive in that they follow on the heels of opinion pieces we have published in the “mSphere of Influence” series. In that series, young microbiologists explained how specific research papers or events provided a context that shaped their ideas. Our authors also presented a brief view of the horizon and how their research or professional directions may unfold. With the Full Circle articles, our authors look back to see how these areas really did unfold!

We are excited to host a series that provides some historical insight into diverse research and professional topics while at the same time giving a voice to young scientists in our field. The series will also help to capture how serendipity can reorient research directions. Our hope is that the whole—the combination of mSpheres of Influence and Full Circles—will be greater than the sum of the parts by allowing readers to trace the impact of ideas and events. Perhaps the most important lesson will be that there are many paths by which ideas develop in microbiology.

